# Dr. John W. Dee

**Published:** 1914-09

**Authors:** 


Dr. John W. Dee, Buffalo, 1869, died at Niagara Falls, December 2, 1913, aged 80.

